# Efficacy of repetitive transcranial magnetic stimulation for subjective chronic tinnitus: a randomized controlled trial meta-analysis

**DOI:** 10.3389/fnins.2025.1579846

**Published:** 2025-04-15

**Authors:** Ziyan He, Defu Liao, Qipei Ji, Shichang Yan, Shuangchun Ai

**Affiliations:** ^1^College of Health and Rehabilitation, Chengdu University of Traditional Chinese Medicine, Chengdu, China; ^2^Department of Rehabilitation Medicine, Mianyang Hospital, Chengdu University of Traditional Chinese Medicine, Mianyang, Sichuan, China

**Keywords:** repetitive transcranial magnetic stimulation, rTMS, chronic tinnitus, meta-analysis, randomized controlled trial

## Abstract

**Objectives:**

Chronic tinnitus affects approximately 10%–15% of the population. The long-term presence of severe tinnitus significantly impacts affected individuals’ quality of life and emotional state. Repetitive transcranial magnetic stimulation (rTMS) is a non-invasive technique that employs pulsed magnetic fields to modulate neural activity. rTMS is considered a promising treatment strategy for chronic tinnitus. However, the therapeutic effect of rTMS on subjective chronic tinnitus remains inconclusive, and its validity is still a subject of debate among researchers.

**Methods:**

To identify RCTs investigating rTMS for subjective chronic tinnitus, a comprehensive computerized search was conducted across multiple databases, including PubMed, The Cochrane Library, Embase, Web of Science, China Knowledge, WIPO, Wanfang, and the China Biomedical Literature Database (CBM). The search timeframe spanned from the inception of each database to 2 June 2024. Two independent investigators performed literature screening, data extraction, and quality assessment using the Cochrane Risk of Bias Assessment Tool. Meta-analysis was conducted using RevMan 5.4 software.

**Results:**

Sixteen randomized controlled trials (RCTs) involving 1,105 chronic tinnitus patients were included. RTMS was superior to Sham rTMS in THI and VAS and had a positive effect on the short-term impact of THI (1 month), Still, this meta-analysis did not observe a positive effect of rTMS on the long-term implications of tinnitus (6 months). rTMS had no significant immediate effect on TQ and LM scores on tinnitus questionnaires.

**Conclusion:**

This meta-analysis demonstrated that rTMS has some efficacy in chronic tinnitus. However, more RCTs are needed to validate its effectiveness, to support the effectiveness of repetitive transcranial magnetic stimulation for tinnitus with larger sample sizes and more follow-up data, and to explore the potential benefits of rTMS in chronic tinnitus.

**Systematic review registration:**

https://www.crd.york.ac.uk/PROSPERO/, identifier CRD42024569403.

## Introduction

Tinnitus is defined as the perception of sound in the absence of corresponding external acoustic stimuli. Unlike auditory hallucinations, which primarily occur in patients with psychiatric disorders and involve the perception of voices or music, tinnitus sensations are typically characterized by unformed acoustic qualities, such as buzzing, and hissing. When the sound is generated within the body and can also be heard by the examiner, it is classified as objective tinnitus. In contrast, subjective tinnitus lacks a specific internal acoustic source. In this study, the authors focus on subjective chronic tinnitus (Langguth et al., 2013). Jarach et al. (2022) reported a pooled prevalence of 14.4% for tinnitus in adults, with a severe tinnitus prevalence of 2.3% of the population. Globally, tinnitus affects more than 740 million adults, with over 120 million considering it a major health concern. Chronic tinnitus often causes emotional disturbances, such as anxiety, depression, sleep disorders, and there can even be problems like hearing loss and cognitive impairment (Caspary and Llano, 2017; Mazurek et al., 2023). This indicates that tinnitus is a global health concern, similar to chronic pain, with a significant lack of effective treatment options.

The pathophysiology underlying tinnitus remains incompletely understood, involving various model theories. Langguth et al. (2024) summarized four principal models - peripheral, central, gate control, and somatosensory model, revealing the abnormal electrical activity of neurons in peripheral and central auditory pathways (including the cerebral cortex). The pathology of chronic tinnitus likely involves multiple systems, often implicating complex neural networks across various brain regions (Hu et al., 2021).

The clinical management of chronic subjective tinnitus primarily involves pharmacological, cognitive behavioral therapy (CBT), neural stimulation, counseling and psychoeducation, tinnitus retraining therapy (TRT), Hearing aids, among other modalities (Langguth et al., 2023). These therapeutic approaches demonstrate varying degrees of efficacy in tinnitus management, particularly guideline-recommended CBT and hearing aid (therapy with concomitant hearing loss) (Langguth et al., 2023). However, these therapies are not satisfactory for all tinnitus patients, so there is an urgent need to find more effective therapeutic strategies for chronic tinnitus.

Repetitive transcranial magnetic stimulation (rTMS) is a non-invasive neuroregulatory technique based on electromagnetic induction. It generates transient magnetic fields via high-intensity pulsed current coils, inducing depolarization of cortical neurons through the scalp and skull. This process modulates the excitability of tinnitus-related neurons and neurotransmitters and enhances the plasticity of auditory neurons (May et al., 2007). The stimulation frequency employed in rTMS is determined by the intended therapeutic outcome. Low-frequency rTMS (= 1 Hz) has been associated with inhibitory effects on neuroplasticity (To et al., 2018). RCTs investigating rTMS for tinnitus have utilized low-frequency stimulation, to reduce neuronal hyperactivity in non-auditory regions implicated in tinnitus pathogenesis. Concurrently, most RCTs have focused on targeting specifically the dorsomedial prefrontal cortex (DMPFC), auditory cortex (AC), or temporoparietal regions at 1 Hz stimulation, demonstrating that the low-frequency rTMS paradigm represents the most widely adopted therapeutic protocol (Denton et al., 2021). Additionally, rTMS offers several advantages, including being non-invasive, painless, and safe. A meta-analysis by Liang et al. (2020) reported that rTMS for tinnitus are a safe option, as severe adverse events were evenly distributed between participants randomized to rTMS and sham rTMS groups. Meng et al. (2011) also supported this view, indicating that rTMS is a safe treatment for tinnitus in the short term, although data supporting its long-term safety are currently lacking. The clinical practice guideline for tinnitus published by the American Academy of Otolaryngology-Head and Neck Surgery assigns a Grade C recommendation to rTMS for treating chronic tinnitus (Lefaucheur et al., 2014). However, the results and conclusions of clinical trials investigating rTMS for tinnitus are varied. Although studies have reported the clinical efficacy and safety of rTMS for chronic tinnitus, the findings remain inconsistent. A survey by Piccirillo et al. (2011) reported that daily low-frequency rTMS applied to the left temporoparietal junction for 2 weeks were no more effective than a placebo in patients with chronic tinnitus. A possible explanation for this result is the short duration of treatment, which may have limited the ability of rTMS to influence the auditory cortex buried within the lateral sulcus. Therefore, this study conducted a meta-analysis by collecting relevant randomized controlled trials (RCTs) to evaluate the efficacy of rTMS for subjective chronic tinnitus.

## Methods

This systematic review and meta-analysis adheres to standardized measurement tools for evaluating systematic reviews and follows the Preferred Reporting Items for Systematic Reviews and Meta-Analyses (PRISMA) guidelines. The study protocol is registered with the International Prospective Register of Systematic Reviews (PROSPERO) under CRD42024569403.

### Inclusion and exclusion criteria

The inclusion criteria are outlined below:

1) Study design: Randomized controlled trials (RCTs) or clinical controlled trials (CCTs).
2) Participants: Patients diagnosed with subjective chronic tinnitus, with a duration of = 6 months, regardless of nationality, race, or age.
3) Intervention: The test group received repetitive transcranial magnetic stimulation (rTMS), while the control group underwent sham stimulation.
4) Primary outcome measure: Tinnitus Handicap Inventory (THI), Secondary outcome measure: Visual Analog Scale (VAS), Tinnitus Loudness Match (LM), and Tinnitus Questionnaire (TQ).

The exclusion criteria are as follows:

1) Conference papers, dissertations, systematic reviews, meta-analyses, animal studies, or studies irrelevant to the research topic.
2) Studies combining rTMS with other interventions (e.g., electroacupuncture) or pharmacological treatments.
3) Studies with incomplete data, uncontactable authors, or unavailable full-text articles.
4) Non-Chinese or non-English publications and duplicate records.

### Database and search

We systematically searched PubMed, The Cochrane Library, Embase, Web of Science, China National Knowledge Infrastructure (CNKI), WIPO, Wanfang, and the China Biomedical Literature Database (CBM) to identify relevant randomized controlled trials (RCTs) on repetitive transcranial magnetic stimulation (rTMS) for the treatment of subjective chronic tinnitus. The search timeframe spanned from database inception to 2 June 2024, utilizing a combination of subject headings and free-text terms. The search keywords included tinnitus, chronic tinnitus, subjective tinnitus, repetitive transcranial magnetic stimulation (rTMS). The detailed search strategy is provided in Table 1.

**TABLE 1:** Basic characteristics of the included studies.

Literature	Study design	Sample size	Age (years)	Course of disease	Treatments	Intervention	Stimulus site
Landgrebe et al., 2017	Randomized double-blind controlled trial	E:71 C:75	E: 48.1 ± 12.5 C: 49.9 ± 13.2	E: 6.2 ± 5.3 years C: 8.1 ± 8.4 years	2 weeks	E: 1 HZ stimulation C: Sham Stimulus	Left temporoparietal cortex
Dai et al., 2018	Randomized controlled trial	E:70 C:70	E: 68.47 ± 3.61 C: 68.13 ± 3.53	E:17.83 ± 2.48 months C:17.46 ± 2.54 months	10 days	E:1 HZ stimulation C:Sham Stimulus	Bilateral temporoparietal cortex
Li et al., 2023	Randomized double- blind controlled trial	E:40 C:20	E:45.975 ± 10.9 C:44.85 ± 11.82	E:9.935 ± 2.69 months C: 9.43 ± 2.70 months	2 weeks	E: 1 HZ stimulation C:Sham Stimulus	Auditory cortex/dorsolateral left prefrontal lobe
Marcondes et al., 2010	Randomized double-blind controlled trial	E:10 C:9	E: ¿18 C: ¿18	NR	1 months	E: 1 HZ stimulation C: Sham Stimulus	Left temporoparietal cortex
Aydın et al., 2021	Randomized double-blind controlled trial	E:40 C:22	E: 49.47 ± 14.16 C: 49.31 ± 12.72	E:24.71 ± 19.99 months C:29.15 ± 28.53 months	5 days	E: 1 HZ stimulation C:Sham Stimulus	Left temporoparietal cortex
Chung et al., 2012	Randomized controlled trial	E:12 C:10	E: 53.83 ± 18.4 C: 51.9 ± 15.5	E: 7.25 ± 7.3 years C: 5.75 ± 2.8 years	1 weeks	E: Continuous pulse 5 HZstimulation C: Sham Stimulus	Auditory cortexin/ the temporoparietal area
Dai et al., 2021	Randomized controlled trial	E:80 C:40	E:49.885 ± 3.35 C: 49.96 ± 3.32	E: 5.12 ± 1.435 years C: 5.65 ± 1.86 years	3 months	E:1 HZ stimulation C: Sham Stimulus	Bilateral temporoparietal cortex/ Stimulation of right dorsolateral/ prefrontal cortex
Ma et al., 2014	Randomized controlled trial	E:15 C:15	E: 49.53 ± 3.08 C: 46.63 ± 6.25	E: 20.8 ± 13.43 months C: 23.7 ± 15.03 months	10 days	E: 1 HZ stimulation C: Sham Stimulus	Tinnitus ipsilateral temporoparietal cortex
Yilmaz et al., 2014	Randomized double-blind controlled trial	E:30 C:30	E: 49.8 ± 8.03 C: 49.8 ± 8.03	NR	1 month	E: 1 HZ stimulation C: Sham Stimulus	Auditory cortex
Hoekstra et al., 2013	Randomized double-blind controlled trial	E:26 C:24	E:50 ± 12 C:50 ± 12	E:46 (8–420) months C:46 (8–420) months	5 days	E:1 HZ stimulation C:Sham Stimulus	Unilateral AC
Kyong et al., 2019	Randomized double-blind controlled trial	E:17 C:13	E:53.6 ± 11.4 C:53.6 ± 11.4	E:76.1 ± 129.3 months C:70.1 ± 70.4 months	4 days	E:1 HZ stimulation C: Sham Stimulus	The left primary AC/left dorsolateral prefrontal cortex
Sahlsten et al., 2017	Randomized Single-blind controlled trial	E:19 C:20	E:48.9 ± 13.1 C:51.5 ± 10.7	E:5.4 ± 2.5 years C:4.9 ± 2.7 years	10 days	E:1 HZ stimulation C:Sham Stimulus	Left auditory cortex
Langguth et al., 2014	Randomized controlled parallel double-blind	E:48 C:45	E:44.9 ± 11.5 C:50.3 ± 12.9	E:68.0 ± 97 C:74.4 ± 74.2	10 days	E:1 HZ stimulation C: Sham Stimulus	PET-based neuronavigation
Langguth et al., 2014	Randomized controlled parallel double-blind	E:47 C:48	E:50.4 ± 12.5 C:50.3 ± 12.9	E:78.3 ± 64.9 C:78.3 ± 64.9	10 days	E:1 HZ stimulation C: Sham Stimulus	Left auditory cortex
Folmer et al., 2015	Randomized Single-blind controlled trial	E:32 C:32	E: 58.3 ± 9.5 C:62.8 ± 8.3	E: > 12 months C: > 12 months	10 days	E:1 HZ stimulation C: Sham Stimulus	Left or right AC
Formánek et al., 2018	Randomized Single-blind controlled trial	E:20 C:12	E:47.9 ± 14.31 C:51.8 ± 10.34	E:53.4 ± 61.89 C:76.8 ± 76.85	10 days	E:1 HZ stimulation C: Sham Stimulus	Left dorsolateral prefrontal cortex
Godbehere et al., 2019	Randomized Single-blind controlled trial	E:23 C:20	NR	NR	5 days	E:5 HZ stimulation C: Sham Stimulus	Temporal-parietal region of the scalp

E, observation group; C, control group; NR, no.

### Studies selection

Two researchers (He and Liao) independently performed literature screening and data extraction using EndNote X9 software. After removing duplicates, animal studies, and review articles, non-compliant literature was excluded based on title and abstract screening, and the predefined inclusion and exclusion criteria were used to conduct the final selection. Full-text articles requiring further evaluation were assessed and selected for inclusion in the analysis.

### Data extraction

Two researchers (He and Liao) independently extracted data using a pre-designed extraction table and cross-checked the following information:

1) Basic information: First author’s name, publication year, sample size, age, disease duration, and stimulation site;
2) Baseline characteristics, intervention details, and pre-and post-treatment outcome data for participants;
3) Primary and secondary outcome measures;
4) Risk of bias assessment: Randomization method, allocation concealment, and other relevant criteria;

Any discrepancies were resolved through consensus or consultation with a third reviewer.

### Risk of bias assessment

The quality of each study was independently assessed by two investigators (He and Liao) using the Cochrane Handbook, which evaluates six key dimensions (Jørgensen et al., 2016):

1) Randomization of the allocation method;
2) Concealment of the allocation scheme;
3) Blinding of participants, treatment administrators, and outcome assessors;
4) Completeness of outcome data;
5) Selective reporting of results;
6) Other potential sources of bias (e.g., small sample size, imbalanced baseline characteristics).

### Data analysis

The study characteristics and findings of the included literature were summarized and presented in tabular form. The results of the risk of bias and reporting quality assessments were summarized using diagrams and tables. Meta-analysis was conducted using Revman5.4 and Stata14 software, and raw effect sizes were pooled. For continuous variables, the mean difference (MD) was used as the effect measure, with each effect size reported along with its 95% confidence interval (CI). A fixed-effects model was applied when *P* > 0.05 and I^2^ < 50%, whereas a random-effects model was employed for results with high heterogeneity (*P* < 0.05 and I^2^ = 50%). In cases of significant heterogeneity, subgroup or sensitivity analyses were conducted to identify its source, and Egger’s test was performed to assess publication bias.

## Results

**FIGURE 1:**
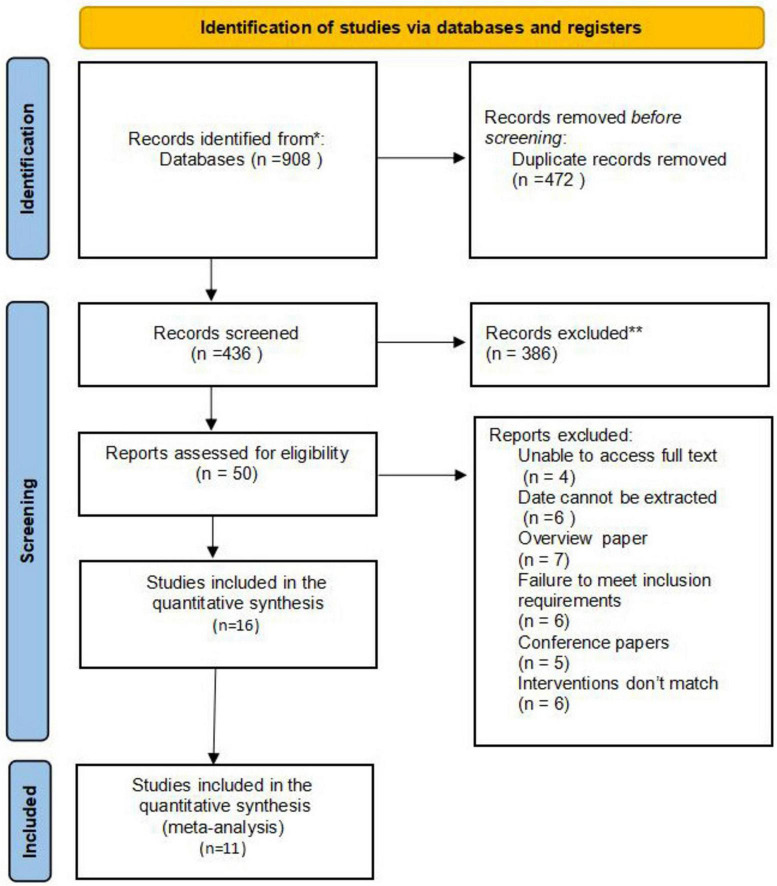
Flowchart of literature screening.

Sixteen randomized controlled trials (RCTs) involving 1,105 participants were identified from 908 publications. The databases searched and the number of records identified in each database were as follows: PubMed (*n* = 325), The Cochrane Library (*n* = 130), Web of Science (*n* = 170), China Biomedical Literature Database (CBM; *n* = 38), China National Knowledge Infrastructure (CNKI; *n* = 36), WanFang Data (*n* = 0), VIP (*n* = 25), and Embase (*n* = 184). A total of 908 potentially relevant articles were identified. After removing duplicates and conducting initial screening, 472 articles were excluded, and 50 full-text articles were retrieved for further assessment. Ultimately, 16 articles were included for analysis. Figure 1 illustrates the study selection process through a PRISMA flow diagram.

### Study characteristics

Twelve English-language studies (Marcondes et al., 2010; Chung et al., 2012; Hoekstra et al., 2013; Langguth et al., 2014; Yilmaz et al., 2014; Folmer et al., 2015; Landgrebe et al., 2017; Sahlsten et al., 2017; Formánek et al., 2018; Godbehere et al., 2019; Kyong et al., 2019; Aydın et al., 2021) and four Chinese-language studies (Ma et al., 2014; Dai et al., 2018; Dai et al., 2021; Li et al., 2023) were ultimately included. The study included 1,105 participants, 600 in the rTMS group and 505 in the Sham group. The participants’ ages ranged from 19 to 73. Fourteen of the 16 studies applied low-frequency stimulation (1 Hz), while two utilized high-frequency stimulation (5 Hz). Twelve studies applied single-site rTMS stimulation; five targeted the temporoparietal cortex, six focused on the auditory cortex, and one study targeted the left dorsolateral prefrontal cortex. Four studies employed multi-site rTMS stimulation, targeting the left temporal cortex combined with the left dorsolateral prefrontal cortex, the bilateral temporoparietal cortex, the left primary AC with the left dorsolateral prefrontal cortex, and the bilateral temporoparietal cortex combined with the right dorsolateral prefrontal cortex. The detailed characteristics of the included studies are summarized in Table 1.

### Risk of bias evaluation results

**FIGURE 2:**
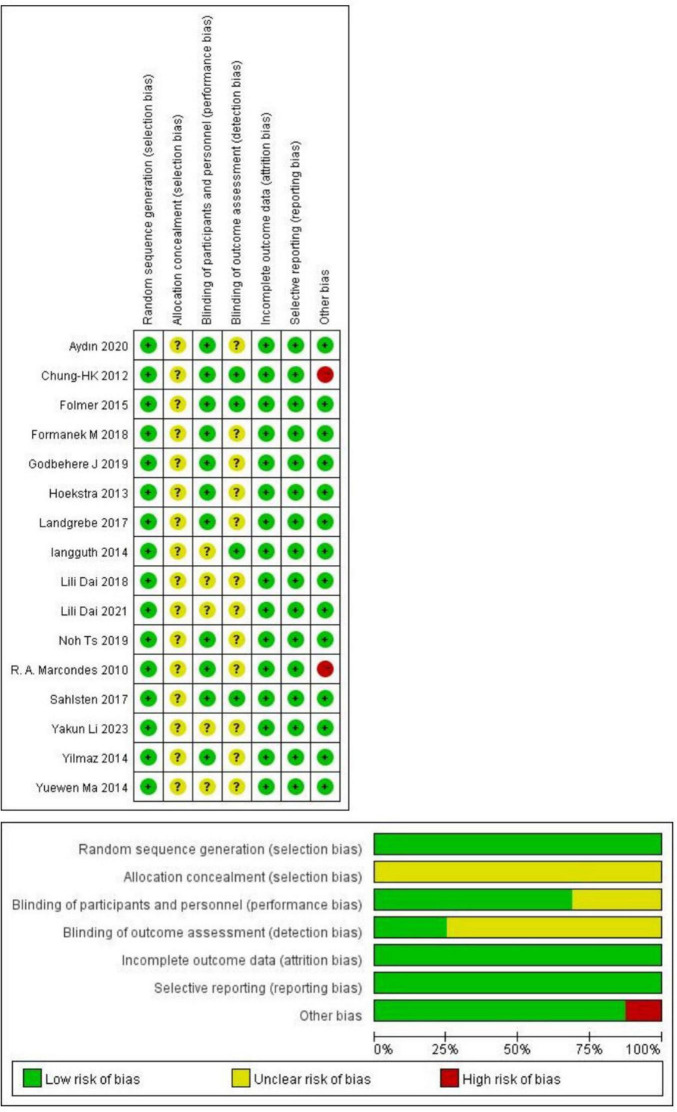
Risk of bias for included studies.

Figure 2 presents the risk of bias assessment results for the included studies. All 16 studies reported using a randomization method. Among these, eight studies (Hoekstra et al., 2013; Langguth et al., 2014; Ma et al., 2014; Yilmaz et al., 2014; Landgrebe et al., 2017; Dai et al., 2018; Aydın et al., 2021; Li et al., 2023) specified the randomization technique, which included random number generation, coin tossing, simple random sampling, and computer-generated sequences. The remaining eight studies (Marcondes et al., 2010; Chung et al., 2012; Folmer et al., 2015; Sahlsten et al., 2017; Formánek et al., 2018; Godbehere et al., 2019; Kyong et al., 2019; Dai et al., 2021) did not describe their randomization methods in detail. Fifteen studies failed to describe allocation concealment and were therefore rated as having a medium risk of bias. Ten studies employed a double-blind design, three used a single-blind approach, and the remaining did not report blinding procedures. All studies adequately reported the completeness of outcome data and demonstrated minimal selective reporting bias. Regarding other sources of bias, 14 were rated as low risk, while the remaining two were classified as high risk due to small sample sizes.

### Meta-analysis

#### Tinnitus Handicap Inventory score

**FIGURE 3:**
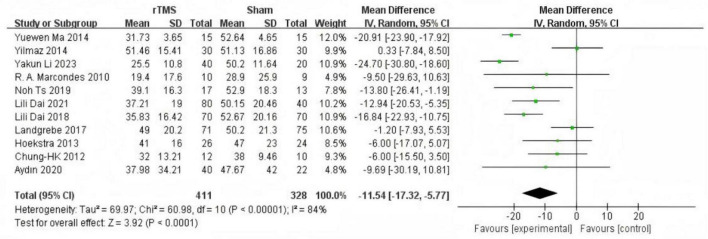
Forest plot: effect of repetitive transcranial magnetic stimulation (rTMS) reduction of Tinnitus Handicap Inventory (THI) score compared with the control group.

Of the 11 included studies (Marcondes et al., 2010; Chung et al., 2012; Hoekstra et al., 2013; Ma et al., 2014; Yilmaz et al., 2014; Landgrebe et al., 2017; Dai et al., 2018; Kyong et al., 2019; Aydın et al., 2021; Dai et al., 2021; Li et al., 2023) involving 739 participants with subjective chronic tinnitus assessed outcomes using the Tinnitus Handicap Inventory (THI). Meta-analysis of these studies using a random-effects model demonstrated that repetitive transcranial magnetic stimulation (rTMS) significantly reduced the adverse impact of tinnitus on physical functioning, mental health, and emotional wellbeing (MD = −11.54, 95% CI (−17.32, −5.77), *P* < 0.00001; Figure 3).

**FIGURE 4:**
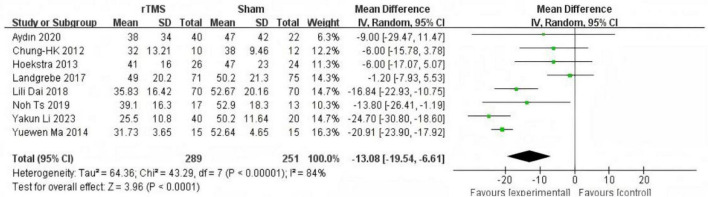
Forest plot: effect of repetitive transcranial magnetic stimulation (rTMS) treatment cycles were less than 1 month Tinnitus Handicap Inventory (THI) score compared with the control group.

**FIGURE 5:**
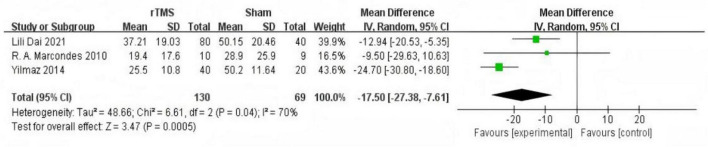
Forest plot: effect of repetitive transcranial magnetic stimulation (rTMS) treatment cycles were more than 1 month Tinnitus Handicap Inventory (THI) score compared with the control group.

Subgroup analysis based on intervention duration included eight studies (Chung et al., 2012; Hoekstra et al., 2013; Ma et al., 2014; Landgrebe et al., 2017; Dai et al., 2018; Kyong et al., 2019; Aydın et al., 2021; Li et al., 2023) with rTMS treatment durations of less than 1 month. Meta-analysis using a random-effects model demonstrated significantly lower THI scores in the experimental group compared to the control group (MD = −13.08, 95% CI (−19.54, −6.61), *P* < 0.0001 Figure 4). Three studies (Marcondes et al., 2010; Yilmaz et al., 2014; Dai et al., 2021) with rTMS treatment durations of 1 month or longer were analyzed using a random-effects model, revealing significantly lower THI scores in the experimental group compared to the control group (MD = −17.50, 95% CI (−27.38, −7.61), *P* = 0.0005 Figure 5).

#### Tinnitus Handicap Inventory score follow-up after treatment

**FIGURE 6:**
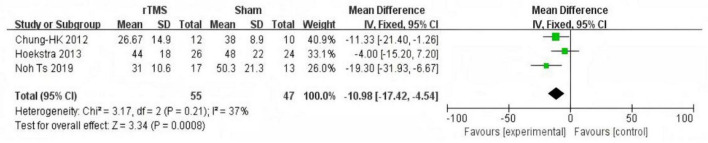
Forest plot: effect of repetitive transcranial magnetic stimulation (rTMS) Mid-term Follow-up Tinnitus Handicap Inventory (THI) score compared with the control group.

**FIGURE 7:**
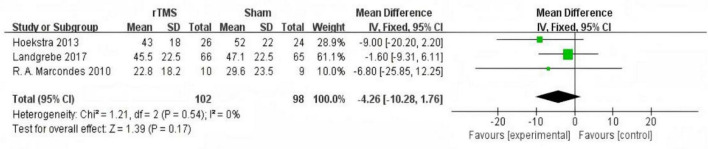
Forest plot: effect of repetitive transcranial magnetic stimulation (rTMS) long-term follow-up Tinnitus Handicap Inventory (THI) score compared with the control group.

Three studies (Chung et al., 2012; Hoekstra et al., 2013; Kyong et al., 2019) provided data on changes in THI scores in the medium-term follow-up (1 month after treatment). The fixed effect model used to summarize and analyze the data showed significant improvement in THI scores in the rTMS group compared with sham stimulation (MD = −10.98, 95% CI (−17.42, −4.54), *P* = 0.0008 Figure 6). Three studies (Marcondes et al., 2010; Hoekstra et al., 2013; Landgrebe et al., 2017) provided data on changes in THI scores in the long-term follow-up (6 months after treatment). The fixed effect model used to summarize and analyze the data showed no significant difference in THI scores between the two groups (MD = −4.26, 95% CI (−10.28, 1.76), *P* = 0.17 Figure 7).

#### Other indicators for outcome evaluation

A meta-analysis was conducted to evaluate patient outcomes across the included studies: four studies (Ma et al., 2014; Dai et al., 2018; Dai et al., 2021; Li et al., 2023) assessed post-intervention VAS scores, three studies (Chung et al., 2012; Hoekstra et al., 2013; Landgrebe et al., 2017) evaluated post-intervention TQ scores, and three studies (Chung et al., 2012; Yilmaz et al., 2014; Aydın et al., 2021) measured post-intervention LM scores. A statistically significant difference in post-intervention VAS scores was observed between the rTMS group and the sham rTMS group (MD = −1.85, 95% CI (−2.26, −1.45), *P* < 0.05). No statistically significant difference in outcome was found between the other two groups (Table 2).

**TABLE 2:** Meta-analysis results of other indicators for outcome evaluation.

Outcomes	Included studies (*n*)	Patients (E/C, n)	Heterogeneity	MD (95% CI)	*P*
VAS score post-intervention	4	205/145	*P* = 0.85;I^2^ = 0%	−1.85 (−2.26, −1.45)	*P* < 0.0001
TQ score post-intervention	3	109/109	*P* = 0.15;I^2^ = 47%	−2.96 (−6.69, 1.57)	*P* = 0.22
LM score post-intervention	3	82/62	*P* = 0.79;I^2^ = 0%	−1.62 (−7.19, 3.94)	*P* = 0.57

VAS, visual analog scale; TQ, tinnitus questionnaire; LM, Loudness Matching Tinnitus.

#### Adverse events

**FIGURE 8:**
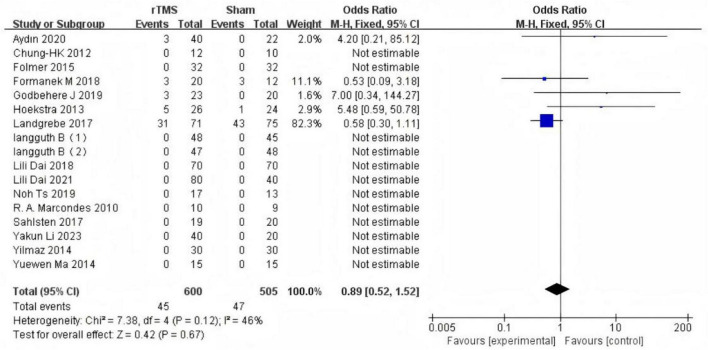
Forest plot: analysis of advance events.

Five studies (Hoekstra et al., 2013; Landgrebe et al., 2017; Formánek et al., 2018; Godbehere et al., 2019; Aydın et al., 2021) reported adverse events following rTMS treatment, involving a total of 92 participants (45 in the rTMS group and 47 in the sham group). A fixed-effects model was employed for the meta-analysis. The results indicated no statistically significant difference in the incidence of adverse events between the rTMS group and the sham-rTMS group (7.5% vs. 9.3%; OR: 0.89, 95% CI: 0.52–1.52; *P* = 0.67) (Figure 8). The most commonly reported symptom of the adverse events was headache. Additionally, three patients experienced facial muscle discomfort, one reported dizziness, one experienced blurred vision, and one described a “battery-licking” sensation (Table 3).

**TABLE 3:** Summary of adverse events.

Literature	Adverse events
Landgrebe et al., 2017	Adverse events were reported in 31 patients in the rTMS group and 43 patients in the sham group. Most adverse events were mild to moderate in severity. Patients in the sham group reported more adverse events than patients in the real rTMS group. More patients in the sham rTMS group complained about deterioration of their tinnitus compared to the real rTMS group. In both groups, headache was the most frequently reported adverse event. In both groups, one Serious adverse event was reported.
Dai et al., 2018	No adverse events
Li et al., 2023	No adverse events
Marcondes et al., 2010	No adverse events
Aydın et al., 2021	A total of three patients in the rTMS group reported temporary localized headaches in the time area, which did not cause much discomfort. The headache disappeared at the end of treatment and no other side effects were reported.
Chung et al., 2012	No adverse events
Dai et al., 2021	No adverse events
Ma et al., 2014	No adverse events
Yilmaz et al., 2014	No adverse events
Hoekstra et al., 2013	Headache was reported by 5 patients in the rTMS group and one patient in the sham surgery group (All five patients had headaches, one of them had dizziness, one also caused a “battery licking” sensation, and one patient in the sham surgery group experienced a placebo (headache) side effect).
Kyong et al., 2019	No adverse events
Sahlsten et al., 2017	No adverse events
Langguth B (1)	No adverse events
Langguth B (2)	No adverse events
Folmer et al., 2015	No adverse events
Formánek et al., 2018	Three patients experienced temporal side effects of rTMS (all headaches) and three patients experienced temporal side effects of placebo (1 headache, 1 dizziness, and 1 blurred vision).
Godbehere et al., 2019	In the rTMS treatment group, three patients withdrew due to secondary stimulation of the facial muscles.

### Sensitivity analysis

**FIGURE 9:**
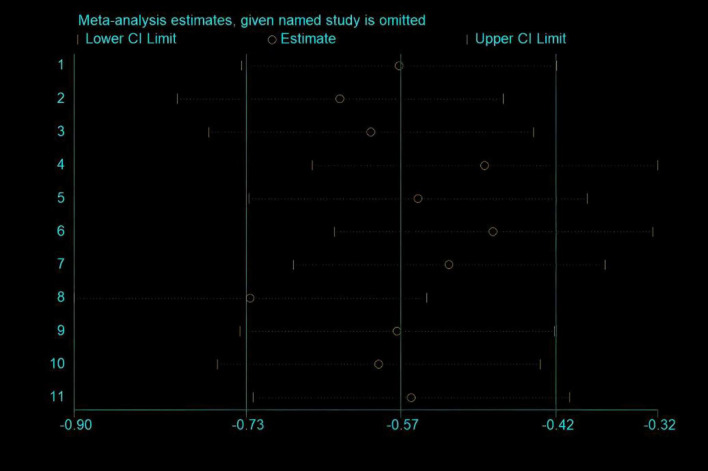
Sensitivity analysis of Tinnitus Handicap Inventory (THI) score.

Due to the high heterogeneity observed in Tinnitus Handicap Inventory (THI) scores, the sensitivity analysis of the included studies revealed that none significantly influenced the overall results (Figure 9).

### Publication bias

Due to the small sample size included, the Egger’s test was performed only on the Tinnitus Handicap Rating Inventory (THI), an outcome indicator, and yielded *P* = 0.34 > 0.05, so it can be judged that there is no significant publication bias in this literature study.

## Discussion

This study presents the results of a systematic review and meta-analysis of 16 randomized controlled trials. To ensure the reliability of our conclusions, we systematically searched, reviewed, and summarized previously published studies on rTMS for chronic subjective tinnitus. Additionally, we conducted a qualitative analysis of adverse events reported across the 16 RCTs to address various clinical questions regarding the efficacy and safety of this treatment. The current meta-analysis revealed that active rTMS significantly impacted the THI and VAS scores in tinnitus patients, with positive effects observed immediately post-treatment and at the 1 month follow-up. However, the findings of this study suggest that the long-term efficacy of active rTMS for tinnitus remains uncertain. Furthermore, the analysis indicated that the direct effects of rTMS on TQ and LM scores were minimal. Previous meta-analyses have reported moderate efficacy of low-frequency rTMS as a treatment for chronic tinnitus, with outcomes assessed using TQ and THI scores. A meta-analysis by Dong et al. (2020) concluded that low-frequency rTMS showed no significant benefit compared to a placebo in chronic tinnitus patients. However, in a study by Schoisswohl et al. (2019), verum rTMS was more effective than sham rTMS. In a systematic review by Yin et al. (2021), THI scores demonstrated positive effects, while TQ scores showed no direct impact, consistent with the findings of the current meta-analysis. Therefore, the use of rTMS as a treatment for tinnitus remains a controversial and unresolved issue. Several factors may have contributed to this result: firstly, treatment parameters on rTMS efficacy are the main correlation. In Schoisswohl et al. (2019) Lower stimulus intensity was significantly associated with the efficacy of rTMS for tinnitus treatment. Secondly, the lower the number of pulses, the better the effect may be. The lengthening of the stimulus does not by itself lead to an improvement in the tinnitus condition. Doubling the length of the stimulus causes reverse aftereffects, such as inhibition becoming excitatory (Gamboa et al., 2010). Finally, differences in the site of rTMS treatment of tinnitus may lead to variability in results. In a study by Watson et al. (2023) it was concluded that the temporoparietal junction is a promising non-auditory rTMS treatment site, with a relatively higher frequency of tinnitus suppression than all other sites. In previous research, the left primary auditory cortex was a potential target for 1 Hz rTMS in tinnitus treatment, as low-frequency rTMS applied to this region can reduce auditory hallucinations in patients (Eichhammer et al., 2003). Thus, the left temporal cortex is the preferred target regardless of which ear is affected.

Based on our findings, rTMS demonstrates a certain level of efficacy in treating chronic tinnitus. Subgroup analysis based on intervention duration revealed that both rTMS groups (treatment duration < 1 month and = 1 month) exhibited lower Tinnitus Handicap Inventory (THI) scores compared to the control group, indicating that both durations can improve chronic tinnitus. However, the effect was more pronounced in the = 1 month group (MD: -17.5, *P* = 0.0005) than in the < 1 month group (MD: -13.08, *P* < 0.0001), suggesting that intervention duration may influence therapeutic outcomes, which is consistent with previous research (Ting et al., 2011). Additionally, data analysis of follow-up outcomes showed that the therapeutic effect was most pronounced immediately post-treatment (MD: -11.54, *P* < 0.00001), gradually diminishing over time (MD: -10.98, *P* = 0.0008), and becoming non-significant at 6 months (MD: -4.26, *P* = 0.17). Therefore, the efficacy of rTMS for tinnitus may be time-limited. Additionally, to assess the safety of rTMS for tinnitus, we conducted a pooled analysis of adverse events. The results indicated that rTMS is a relatively safe option, as adverse events were evenly distributed between the rTMS and sham rTMS groups, consistent with previous findings (Liang et al., 2020).

The heterogeneity observed in some results of this meta-analysis may be attributed to variations in rTMS parameters, such as frequency and duration, as well as differences in patient baseline characteristics, including age, tinnitus severity, or duration. Additionally, regional differences among patients, including lifestyle, cultural background, cognitive factors, and educational level, may also impact the robustness of the results. Another potential factor is the inherent subjectivity of the assessment scales and the limited sample size in some included studies. Therefore, future research should include more extensive, multicenter, randomized controlled trials to further validate the efficacy of rTMS for tinnitus.

This study has several advantages compared to previous meta-analyses on rTMS for tinnitus. First, we included the latest RCTs (Aydın et al., 2021; Dai et al., 2021; Li et al., 2023) in addition to previous studies, and our search encompassed both Chinese and English literature, ensuring comprehensiveness. Additionally, we conducted subgroup analyses based on intervention duration and follow-up time, providing a multi-faceted exploration of the efficacy of rTMS for chronic tinnitus. Furthermore, we performed a series of assessments on the included studies to ensure the high reliability of our conclusions. Second, most of the included studies were single—or double-masked, providing a relatively high level of evidence and enhancing the results’ reliability. Moreover, sensitivity analysis and publication bias assessment were conducted to ensure the stability of this meta-analysis. Finally, we performed a quantitative analysis of the safety of rTMS for chronic tinnitus.

Additionally, it is important to acknowledge several limitations of our study. First, although recent large-scale randomized trials were included, the limited number of participants in our meta-analysis constrained the accuracy of the analysis. Additionally, some results were non-significant, which may be attributed to the heterogeneous nature of the population receiving rTMS. Future research should encourage large-scale, multicenter randomized controlled trials with standardized protocols. Furthermore, exploring combination therapies, such as rTMS with cognitive behavioral therapy (CBT), rTMS combined with prefrontal stimulation, high-frequency protocol, and auditory stimulation may yield synergistic effects.

## Data Availability

The original contributions presented in this study are included in this article/supplementary material, further inquiries can be directed to the corresponding author.

## References

[B1] Aydın M. Erkan M. Gündoğdu R. Vural A. Kökoğlu K. ahin M. (2021). Assessment of the effectiveness of transcranial magnetic stimulation in subjective tinnitus. *Int. Arch. Otorhinolaryngol.* 25 e453–e458. 10.1055/s-0040-1718530 34377184 PMC8321633

[B2] Caspary D. M. Llano D. A. (2017). Auditory thalamic circuits and GABA(a) receptor function: Putative mechanisms in tinnitus pathology. *Hear. Res.* 349 197–207. 10.1016/j.heares.2016.08.009 27553899 PMC5319923

[B3] Chung H. K. Tsai C. H. Lin Y. C. Chen J. M. Tsou Y. A. Wang C. Y. (2012). Effectiveness of theta-burst repetitive transcranial magnetic stimulation for treating chronic tinnitus. *Audiol. Neuro-Otol.* 17 112–120. 10.1159/000330882 21865723

[B4] Dai L. Chen Z. Wu H. Xu Y. (2018). Clinical efficacy of low-frequency repetitive transcranial magnetic stimulation in the treatment of chronic subjective tinnitus in the elderly. *Chin. J. Gerontol.* 38 2950–2952. 10.3969/j.issn.1005-9202.2018.12.054

[B5] Dai L. Tang W. Shuang G. Wu H. Chen Z. (2021). Continuous θ burst stimulation combined low frequency heavy. Clinical analysis of stimulation therapy for subjective tinnitus. *Clin. Educ. General Pract.* 19 432–435. 10.13558/j.cnki.issn1672-3686.2021.005.014

[B6] Denton A. J. Finberg A. Ashman P. E. Bencie N. B. Scaglione T. Kuzbyt B. (2021). Implications of transcranial magnetic stimulation as a treatment modality for tinnitus. *J. Clin. Med.* 10:5422. 10.3390/jcm10225422 34830704 PMC8622674

[B7] Dong C. Chen C. Wang T. Gao C. Wang Y. Guan X. (2020). Low-frequency repetitive transcranial magnetic stimulation for the treatment of chronic tinnitus: A systematic review and meta-analysis of randomized controlled trials. *Biomed. Res. Int.* 2020:3141278. 10.1155/2020/3141278 32461976 PMC7218966

[B8] Eichhammer P. Langguth B. Marienhagen J. Kleinjung T. Hajak G. (2003). Neuronavigated repetitive transcranial magnetic stimulation in patients with tinnitus: A short case series. *Biol. Psychiatry* 54 862–865. 10.1016/s0006-3223(02)01896-6 14550687

[B9] Folmer R. L. Theodoroff S. M. Casiana L. Shi Y. Griest S. Vachhani J. (2015). Repetitive transcranial magnetic stimulation treatment for chronic tinnitus: A randomized clinical trial. *JAMA Otolaryngol. Head Neck Surg.* 141 716–722. 10.1001/jamaoto.2015.1219 26181507

[B10] Formánek M. Migal’ová P. Krulová P. Bar M. Jančatová D. Zakopčanová-Srovnalová H. (2018). Combined transcranial magnetic stimulation in the treatment of chronic tinnitus. *Ann. Clin. Transl. Neurol.* 5 857–864. 10.1002/acn3.587 30009203 PMC6043768

[B11] Gamboa O. L. Antal A. Moliadze V. Paulus W. (2010). Simply longer is not better: Reversal of theta burst after-effect with prolonged stimulation. *Exp. Brain Res.* 204 181–187. 10.1007/s00221-010-2293-4 20567808 PMC2892066

[B12] Godbehere J. Sandhu J. Evans A. Twigg V. Scivill I. Ray J. (2019). Treatment of tinnitus using theta burst based repetitive transcranial magnetic stimulation-a single blinded randomized control trial. *Otol. Neurotol.* 40 S38–S42. 10.1097/MAO.0000000000002207 31225821

[B13] Hoekstra C. E. Versnel H. Neggers S. F. Niesten M. E. van Zanten G. A. (2013). Bilateral low-frequency repetitive transcranial magnetic stimulation of the auditory cortex in tinnitus patients is not effective: A randomised controlled trial. *Audiol. Neuro Otol.* 18 362–373. 10.1159/000354977 24157459

[B14] Hu J. Cui J. Xu J. J. Yin X. Wu Y. Qi J. (2021). The neural mechanisms of tinnitus: A perspective from functional magnetic resonance imaging. *Front. Neurosci.* 15:621145. 10.3389/fnins.2021.621145 33642982 PMC7905063

[B15] Jarach C. M. Lugo A. Scala M. van den Brandt P. A. Cederroth C. R. Odone A. (2022). Global prevalence and incidence of tinnitus: A systematic review and meta-analysis. *JAMA Neurol.* 79 888–900. 10.1001/jamaneurol.2022.2189 35939312 PMC9361184

[B16] Jørgensen L. Paludan-Müller A. S. Laursen D. R. Savović J. Boutron I. Sterne J. A. (2016). Evaluation of the Cochrane tool for assessing risk of bias in randomized clinical trials: Overview of published comments and analysis of user practice in Cochrane and non-Cochrane reviews. *Syst. Rev.* 5:80. 10.1186/s13643-016-0259-8 27160280 PMC4862216

[B17] Kyong J. S. Noh T. S. Park M. K. Oh S. H. Lee J. H. Suh M. W. (2019). Phantom perception of sound and the abnormal cortical inhibition system: An electroencephalography (EEG) study. *Ann. Otol. Rhinol. Laryngol.* 128 84S–95S. 10.1177/0003489419837990 31092043

[B18] Landgrebe M. Hajak G. Wolf S. Padberg F. Klupp P. Fallgatter A. J. (2017). 1-Hz rTMS in the treatment of tinnitus: A sham-controlled, randomized multicenter trial. *Brain Stimul.* 10 1112–1120. 10.1016/j.brs.2017.08.001 28807845

[B19] Langguth B. de Ridder D. Schlee W. Kleinjung T. (2024). Tinnitus: Clinical Insights in its Pathophysiology-a Perspective. *JARO* 25 249–258. 10.1007/s10162-024-00939-0 38532055 PMC11150221

[B20] Langguth B. Kleinjung T. Schlee W. Vanneste S. De Ridder D. (2023). Tinnitus guidelines and their evidence base. *J. Clin. Med.* 12:3087. 10.3390/jcm12093087 37176527 PMC10178961

[B21] Langguth B. Kreuzer P. M. Kleinjung T. De Ridder D. (2013). Tinnitus: Causes and clinical management. *Lancet Neurol.* 12 920–930. 10.1016/S1474-4422(13)70160-1 23948178

[B22] Langguth B. Landgrebe M. Frank E. Schecklmann M. Sand P. G. Vielsmeier V. (2014). Efficacy of different protocols of transcranial magnetic stimulation for the treatment of tinnitus: Pooled analysis of two randomized controlled studies. *World J. Biol. Psychiatry* 15 276–285. 10.3109/15622975.2012.708438 22909265

[B23] Lefaucheur J. P. André-Obadia N. Antal A. Ayache S. S. Baeken C. Benninger D. H. (2014). Evidence-based guidelines on the therapeutic use of repetitive transcranial magnetic stimulation (rTMS). *Clin. Neurophysiol.* 125 2150–2206. 10.1016/j.clinph.2014.05.021 25034472

[B24] Li Y. Cui W. Zhuang S. Shi Z. Xie C. Cai X. (2023). Clinical effect of double-site repetitive transcranial magnetic stimulation on chronic tinnitus. *Henan Med. Res.* 32 988–992. 10.3969/j.issn.1004-437X.2023.06.007

[B25] Liang Z. Yang H. Cheng G. Huang L. Zhang T. Jia H. (2020). Repetitive transcranial magnetic stimulation on chronic tinnitus: A systematic review and meta-analysis. *BMC Psychiatry* 20:547. 10.1186/s12888-020-02947-9 33228598 PMC7684956

[B26] Ma Y. Zhang D. Shen Y. (2014). Observation on the efficacy of low frequency repetitive transcranial magnetic stimulation in the treatment of subjective tinnitus. *Chin. J. Phys. Med. Rehabil.* 36 299–301. 10.3760/cma.j.issn.0254-1424.2014.04.016 30704229

[B27] Marcondes R. A. Sanchez T. G. Kii M. A. Ono C. R. Buchpiguel C. A. Langguth B. (2010). Repetitive transcranial magnetic stimulation improve tinnitus in normal hearing patients: A double-blind controlled, clinical and neuroimaging outcome study. *Eur. J. Neurol.* 17 38–44. 10.1111/j.1468-1331.2009.02730.x 19614962

[B28] May A. Hajak G. Gänssbauer S. Steffens T. Langguth B. Kleinjung T. (2007). Structural brain alterations following 5 days of intervention: Dynamic aspects of neuroplasticity. *Cereb. Cortex.* 17 205–210. 10.1093/cercor/bhj138 16481564

[B29] Mazurek B. Böcking B. Dobel C. Rose M. Brüggemann P. (2023). Tinnitus and influencing comorbidities. *Laryngorhinootologie* 102 S50–S58. 10.1055/a-1950-6149 37130530 PMC10184670

[B30] Meng Z. Liu S. Zheng Y. Phillips J. S. (2011). Repetitive transcranial magnetic stimulation for tinnitus. *Cochrane Database Syst. Rev.* 5:CD007946. 10.1002/14651858.CD007946.pub2 21975776

[B31] Piccirillo J. F. Garcia K. S. Nicklaus J. Pierce K. Burton H. Vlassenko A. G. (2011). Low-frequency repetitive transcranial magnetic stimulation to the temporoparietal junction for tinnitus. *Arch. Otolaryngol. Head Neck Surg.* 137 221–228. 10.1001/archoto.2011.3 21422304 PMC3140874

[B32] Sahlsten H. Virtanen J. Joutsa J. Niinivirta-Joutsa K. Löyttyniemi E. Johansson R. (2017). Electric field-navigated transcranial magnetic stimulation for chronic tinnitus: A randomized, placebo-controlled study. *Int. J. Audiol.* 56 692–700. 10.1080/14992027.2017.1313461 28415897

[B33] Schoisswohl S. Agrawal K. Simoes J. Neff P. Schlee W. Langguth B. (2019). RTMS parameters in tinnitus trials: A systematic review. *Sci. Rep.* 9:12190. 10.1038/s41598-019-48750-9 31434985 PMC6704094

[B34] Ting S. K. Chan Y. M. Cheong P. W. Wong M. Fook-Chong S. Lo Y. L. (2011). Short duration repetitive transcranial magnetic stimulation for tinnitus treatment: A prospective Asian study. *Clin. Neurol. Neurosurg.* 113 556–558. 10.1016/j.clineuro.2011.03.015 21507564

[B35] To W. T. De Ridder D. Hart J. J. Vanneste S. (2018). Changing brain networks through non-invasive neuromodulation. *Front. Hum. Neurosci.* 12:128. 10.3389/fnhum.2018.00128 29706876 PMC5908883

[B36] Watson N. Schaper F. Jabbour S. Sadler S. Bain P. A. Fox M. D. (2023). Is there an optimal repetitive transcranial magnetic stimulation target to treat chronic tinnitus? *Otolaryngol. Head Neck Surg.* 168 300–306. 10.1177/01945998221102082 35671136

[B37] Yilmaz M. Yener M. H. Turgut N. F. Aydin F. Altug T. (2014). Effectiveness of transcranial magnetic stimulation application in treatment of tinnitus. *J. Craniofac. Surg.* 25 1315–1318. 10.1097/SCS.0000000000000782 25006914

[B38] Yin L. Chen X. Lu X. An Y. Zhang T. Yan J. (2021). An updated meta-analysis: Repetitive transcranial magnetic stimulation for treating tinnitus. *J. Int. Med. Res.* 49:1221799101. 10.1177/0300060521999549 33729855 PMC7975580

